# FGF /FGFR Signal Induces Trachea Extension in the *Drosophila* Visual System

**DOI:** 10.1371/journal.pone.0073878

**Published:** 2013-08-26

**Authors:** Wei-Chen Chu, Yuan-Ming Lee, Yi Henry Sun

**Affiliations:** 1 Graduate Institute of Life Sciences, National Defense Medical Center, Taipei, Taiwan; 2 Institute of Molecular Biology, Academia Sinica, Taipei, Taiwan; 3 Department of Life Sciences and Institute of Genome Sciences, National Yang-Ming University, Taipei, Taiwan; Stockholm University, Sweden

## Abstract

The *Drosophila* compound eye is a large sensory organ that places a high demand on oxygen supplied by the tracheal system. Although the development and function of the *Drosophila* visual system has been extensively studied, the development and contribution of its tracheal system has not been systematically examined. To address this issue, we studied the tracheal patterns and developmental process in the *Drosophila* visual system. We found that the retinal tracheae are derived from air sacs in the head, and the ingrowth of retinal trachea begin at mid-pupal stage. The tracheal development has three stages. First, the air sacs form near the optic lobe in 42-47% of pupal development (pd). Second, in 47-52% pd, air sacs extend branches along the base of the retina following a posterior-to-anterior direction and further form the tracheal network under the fenestrated membrane (TNUFM). Third, the TNUFM extend fine branches into the retina following a proximal-to-distal direction after 60% pd. Furthermore, we found that the trachea extension in both retina and TNUFM are dependent on the FGF(Bnl)/FGFR(Btl) signaling. Our results also provided strong evidence that the photoreceptors are the source of the Bnl ligand to guide the trachea ingrowth. Our work is the first systematic study of the tracheal development in the visual system, and also the first study demonstrating the interactions of two well-studied systems: the eye and trachea.

## Introduction

As an organ grows in size, its surface to volume ratio decreases, and simple diffusion through the surface is not sufficient to support the exchange of nutrients, wastes and gases. In vertebrates, the vascular systems form highly branched networks to fulfill these transport needs. In insects, the tracheal system formed by a network of hollow tubes takes care of the gas exchanges by passive diffusion or by active transport during flight [1].

The tracheal system in the *Drosophila* embryo has been extensively studied [2–7]. The embryonic tracheal development begins from the specification of distinct tracheal placodes in the posterior thoracic and abdominal segments by spatial patterning genes. The placode cells express two transcription factors, Trachealess (Trh) and Ventral veinless (Vvl), that together specify the tracheal fate. The tracheal placodes invaginate to form tracheal sacs and these cells undergo one round of mitosis to generate the final number of about 80 cells per tracheal metamere. Further morphogenesis does not involve cell division. Subsets of tracheal cells then migrate along stereotypical directions to form distinct tracheal branches. The migration is dependent on the fibroblast growth factor (FGF) and FGF receptor (FGFR) signaling. All tracheal cells express the FGFR Breathless (Btl), induced by Trh and Vvl. The tracheal cells then migrate toward the source of FGF ligand Branchless (Bnl) expressed from target cells. The migration along distinct pathways also depends on integrin, EGF and Slit/Robo signalings. Adjacent and contralateral tracheal metameres are then connected by specialized fusion cells to form the interconnected tracheal network. The terminal cells can extend long subcellular tubes for close contact with cells in the target tissue. The patterns of primary and secondary branches are controlled by a hard-wired developmental program. In contrast, terminal branches are variable and regulated by the tissue oxygen requirement. Bnl expression is regulated by hypoxia to ensure tracheal structure matches the cellular oxygen requirement [8]. In addition to the target tissue, tracheal cells themselves can also sense hypoxia and regulate Btl expression for the tracheal branch remodeling [9]. Bnl/Btl signaling also regulates cell proliferation and migration of the trachea that associated with the larval wing disc (dorsal air sac primordium or tracheoblast) [10–12]. The larval tracheal system is largely remodeled during metamorphosis [13].

The *Drosophila* compound eye contains 750~800 ommatidia (unit eyes), each composed of eight photoreceptor neurons (R1 ~ R8), four cone cells, two primary pigment cells, in addition to sharing secondary and tertiary pigment cells and the interommatidial bristles with adjacent ommatidia. The axons of photoreceptors project basally through the fenestrated membrane (FM) and terminate at different layers of the optic lobe. The energy metabolism of insect photoreceptor is predominantly aerobic [14], therefore it places a high demand on oxygen supply. It has been shown that function of retina is sensitive to hypoxia in many organisms including human, mice and honeybee [15–17]. Oxygen transport to the visual system is therefore important to support its neuronal activities.

The compound eye develops from the larval eye-antenna imaginal disc, which is composed of two epithelial sheets and does not contain tracheal cells. The photoreceptors begin to differentiate at third instar larval stage and the retina begins to thicken in the mid to late pupal stage. The thickening and increase in volume suggest a requirement for tracheal ingrowth to provide oxygen. Although it has been shown that many insects have tracheae in the retina with different distribution patterns [18–23], the pattern and development of trachea in the compound eye of *Drosophila* is largely unknown. In this study, we examined the tracheal patterns in the *Drosophila* visual system and studied the molecular mechanism for its development.

We generated a 3D reconstruction of the tracheal system in the adult compound eye and optic lobe. We found that the retinal tracheae are derived from air sacs in the head, and the ingrowth of retina trachea begin at mid-pupal stage. There are three major steps for the development of retinal trachea. First, air sacs become apparent near the optic lobe in 42-47% of pupal development (pd). Second, in 47-52% pd, air sacs extend branches along the fenestrated membrane following a posterior-to-anterior direction and further form the tracheal network under the fenestrated membrane (TNUFM). Third, the TNUFM extend fine branches into the retina following a proximal-to-distal direction after 60% pd. Our results showed that the ingrowth of retinal trachea is dependent on FGF(Bnl)/FGFR(Btl) signaling. We also provide strong evidence suggesting that the photoreceptors are the source of the Bnl ligand to guide the trachea ingrowth.

## Materials and Methods

### Fly stocks

All stocks were grown on standard fly food at room temperature or 25°C. Flies used in this study were *w* ^1118^, *Canton-S*, *btl-Gal4* [24], *bnl-Gal4* [25], *trh-lacZ* (1-eve-1) [26,27], *UAS-Bnl* [28], *UAS-mCD8GFP* [29], *UAS-GFP.nls* (Bloomington *Drosophila* stock center, BDSC-4775), *UAS-H2B-RFP* (from Yohanns Bellaiche), *btl-Gal4*, *UAS-GFP; UAS-DsRed* [13], *longGMR-Gal4* [30], *rh1-Gal4* [31], *sca-Gal4* [32], *spa-Gal4* [33], *repo-Gal4* [34], *CG7077-Gal4* (BDSC-24501), *elav-Gal4* [35], *UAS-Bnl-RNAi* (stocks 5730 and 101377 from Vienna *Drosophila* RNAi Center; the two *UAS-Bnl-RNAi* were combined together in order to enhance the knockdown effect), *UAS-Btl* ^*DN*^ [36], *y w ey-ﬂp; FRT82B Ubi-GFP RpS3/TM6B Tb* and *y w; FRT82B bnl* ^00857^ */TM6B Tb* [37], *sev* ^14^ (=*sev* ^*d2*^) [38], *GMR-wIR* (13D) [39], *bnl* ^p2^ [28], *sc^10^, * [40].

### Eye pigment bleaching

**Figure 1:**
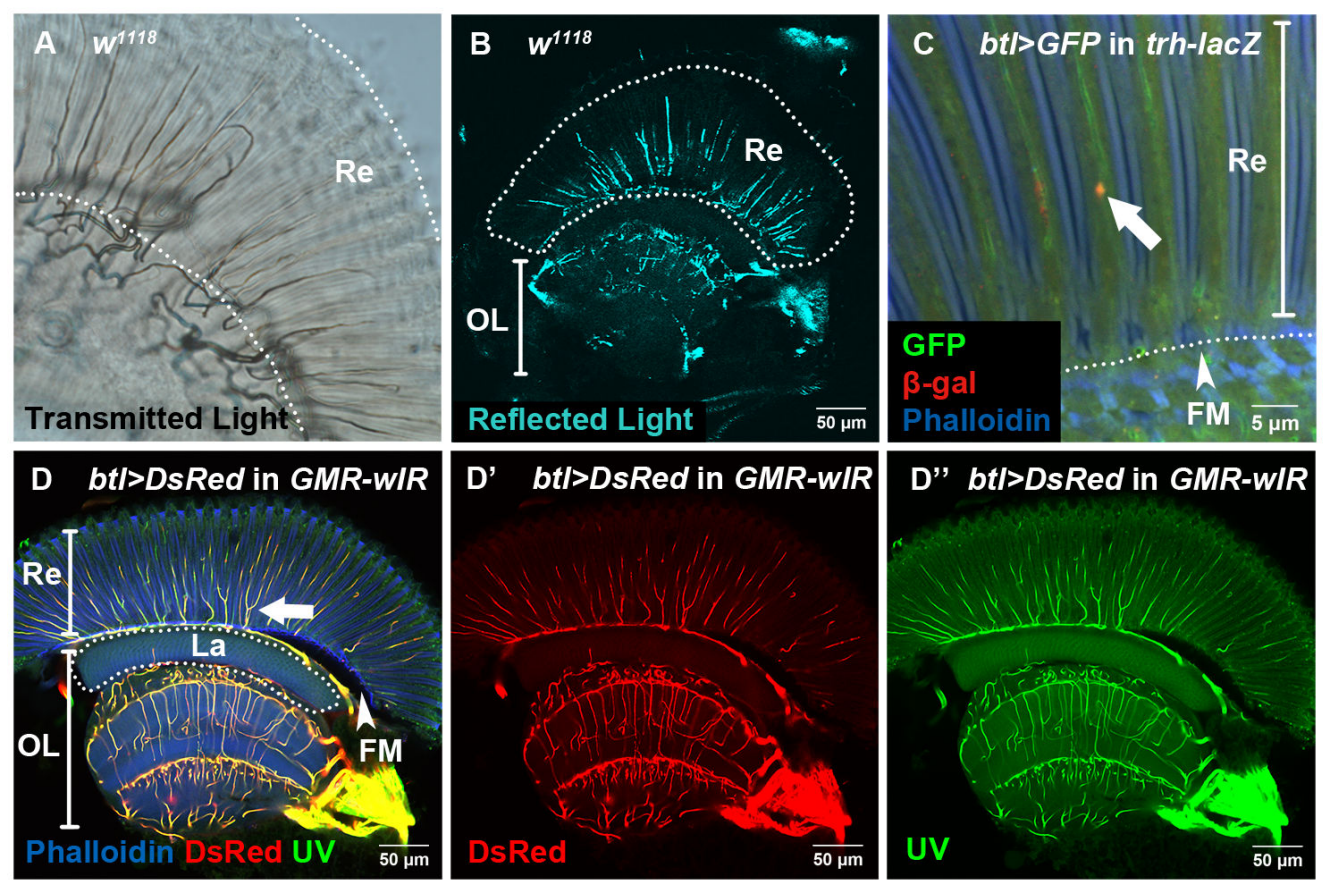
Trachea in the visual system can be observed by multiple methods. (A) The tracheal pattern was observed by transmitted light in the whole-mount dissected *w* ^1118^ adult eye. The dark pattern is due to loss of transmitted light by light reflection of air-filled tubes. The thicker branches near the base of the retina (Re) are connected to thinner branches that extend into the retina. (B) The tracheal pattern was observed by 633 nm reflected light by confocal microscopy in the whole-mount dissected *w* ^1118^ adult eye. (C) The tracheae were visualized by the tracheal markers *btl*>*GFP* (green) and *trh-lacZ* (stained with anti-β-Gal, red). The white arrow points to a nucleus expressing *trh-lacZ*. (D-D”) Visualizing trachea by *btl > DsRed* (red) in *GMR-wIR* and by UV-excited autofluorescence (green). One longitudinal optical section of the adult visual system is shown. The arrow points to a branching tracheal tube in the retina. Re: retina; OL: optic lobe; La: lamina; FM: fenestrated membrane (arrowhead). All images in this and the following figures are based on whole-mount fixed tissues. The sections are confocal optical sections.

**Figure 2:**
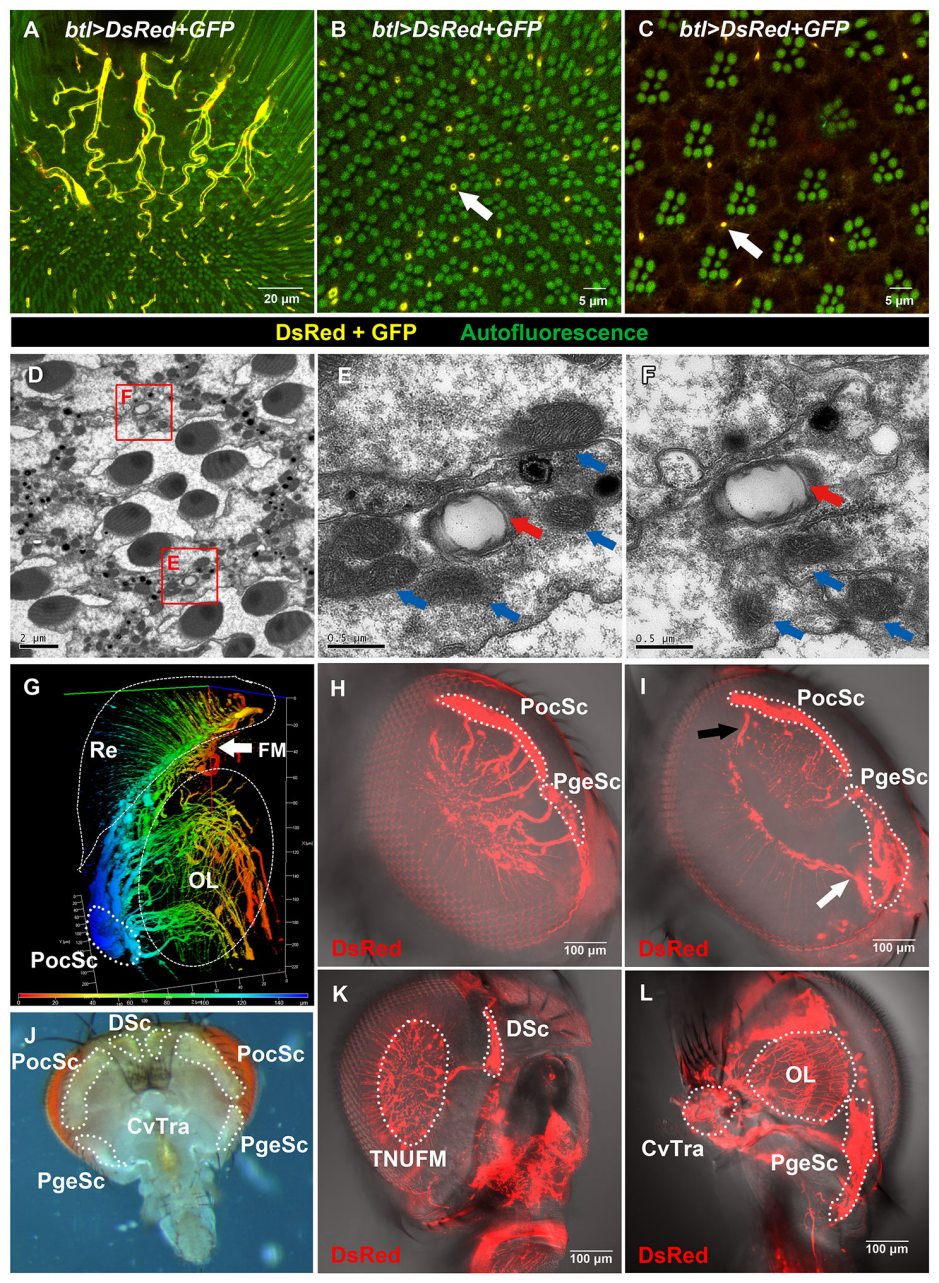
Tracheal patterns in adult eye and its relationship with the air sacs in the head. (A–C) In the optical sections of whole-mount dissected adult retina, the tracheae were labeled by *btl*>DsRed+*GFP* (yellow). Rhabdomeres and fenestrated membrane (FM) were visualized by autofluorescence (green) from the red pigments of the eye. (A) The tracheal network under the fenestrated membrane (TNUFM, thicker tubes) is connected to the retinal tracheae (thinner tubes). (B) At the proximal region of the retina, the tracheal cross section appears donut-shaped (white arrow), indicating the tracheal lumen. (C) At the distal part of retina, the tracheal cross section appears dot-shaped (white arrow). (D–F) Electron microscopy images of the proximal region of the retina. (D) Tracheal lumen can be observed (red boxes, higher magnification in E-F). (E–F) The tracheal characteristic *Taenidium* (ring structure, red arrow), and surrounding mitochondria (blue arrow) can be observed. (G) 3D reconstruction of tracheae in the whole-mount dissected adult visual system (see also Video S2). The tracheae were labeled by *btl > DsRed* in *GMR-wIR*. The pseudocolors indicate the depth from periphery (blue) to center (red). (J) The positions of air sacs in the adult head. (H, I, K, L) The tracheal system (labeled by *btl > DsRed* in *GMR-wIR*) in fixed and FocusClear treated half-head samples (H–I, the same sample at different focal planes). The PocSc extended five major branches to the TNUFM. Four of them are shown in (H) and the fifth branch is shown in (I, black arrow). The PgeSc extended three major branches to the TNUFM. Two of them are shown in (H) and the third branch is shown in (I, white arrow). (K) The DSc extended one major branch to the TNUFM. (L) The tracheae in the optic lobe (OL) were also connected with cervical trachea (CvTra). Re: retina; OL: optic lobe; FM: fenestrated membrane; PocSc: post-ocular sac; PgeSc: post-genal sac; DSc: dorsal sac.

**Figure 3:**
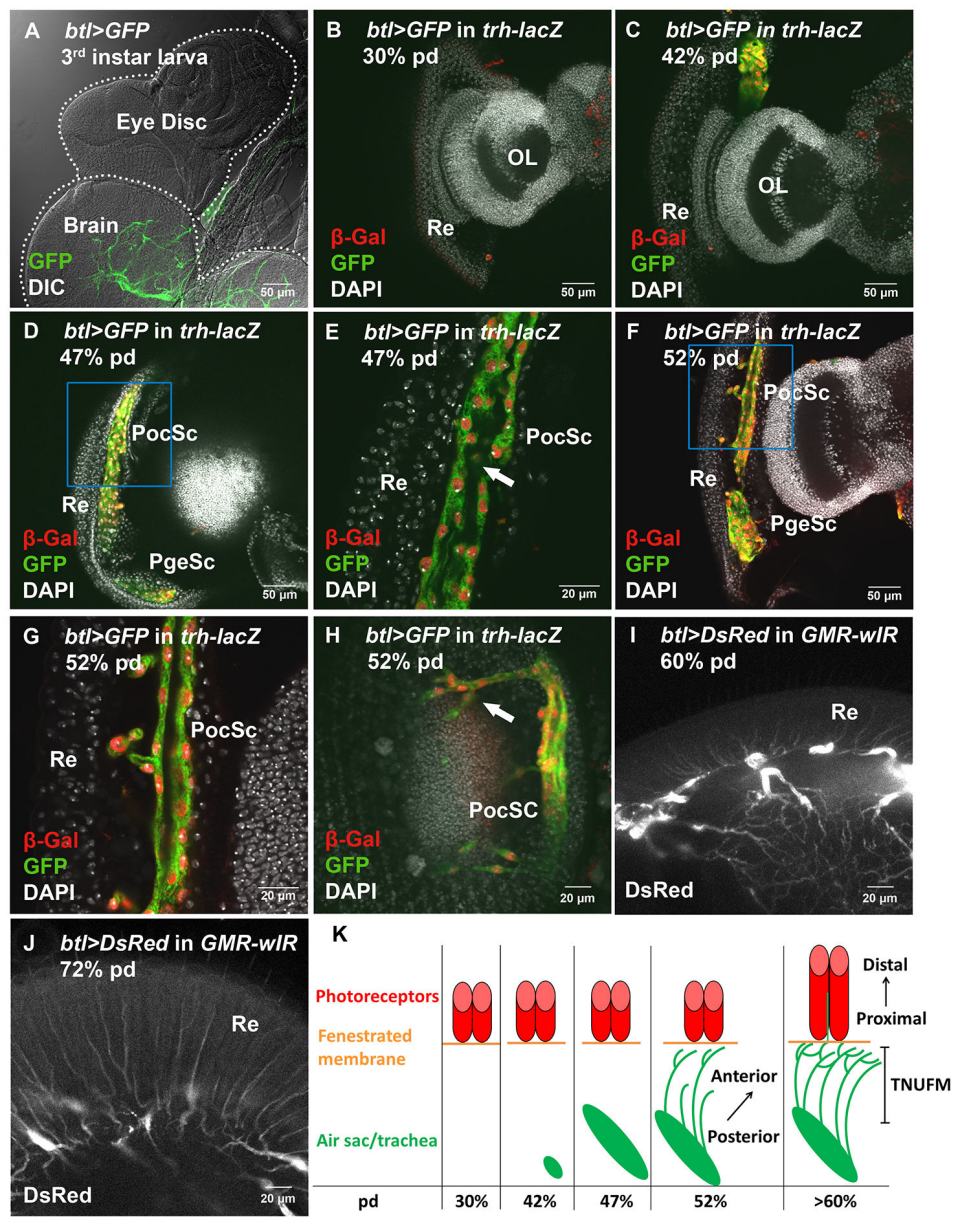
The process of tracheal development in the *Drosophila* visual system. (A) Tracheae labeled by *btl*>*GFP* (green) can be found in brain but not in the eye-antennal disc at third instar larva. (B–H) Tracheal cells were labeled by *btl*>*GFP* (green) and *trh-lacZ* (stained with anti-β-Gal, red). (B) No tracheal cells were found at 30% pd. (C) A group of tracheal cell appeared at 42% pd. (D–E) Air sacs can be found at 47% pd. (D) Two air sacs can be observed, the larger being PocSc and the smaller one being PgeSc (See also Figure 2J). (E) Tracheal lumen (white arrow) can be observed in the PocSc at 47% pd. (F–H) Air sacs extended major branches along the FM. (F) Branches extended from PocSc but not PgeSc at 52% pd. (G) Higher magnification of the inset in (F). (H) Major branches extended from PocSc following the posterior-to-anterior direction at 52% pd. Bifurcating branches can be observed (white arrow). (I–J) The tracheal pattern at later pupal stages were labeled by *btl > DsRed* (white) in *GMR-wIR* in order to decrease the autofluorescence from the red pigments of eye. (I) Ingrowth of fine tracheal branches to retina at 60% pd. (J) Retinal tracheae continue to extend in length following the thickening retina at 72% pd in a proximal-to-distal direction. (K) Schematic representation of the process of tracheal development in the visual system. TNUFM: the tracheal network under the fenestrated membrane. Re: retina; OL: optic lobe; PocSc: post-ocular sac; PgeSc: post-genal sac.

To reduce the strong autofluorescence from the red pigments in the retinal pigment cells, 1% sodium borohydride in phosphate buffered saline (PBS) with 0.3% Triton X-100(PBST) was used [41] (personal communication from Takashi Suzuki). Whole-mount dissected samples were rinsed in 1% sodium borohydride for 1 h at room temperature. Following 10 min washes in PBST three times, the samples were immunostained. For adult and late pupa whole-mount dissected samples (Figure 1D, Figure 2G–I, K-L, Figure 3I-J, Video S2), *GMR-wIR* was included to knocking down *white* (w) expression in retina to reducing the autofluorescence from the retinal pigments.

### Immunostaining and imaging

**Figure 4:**
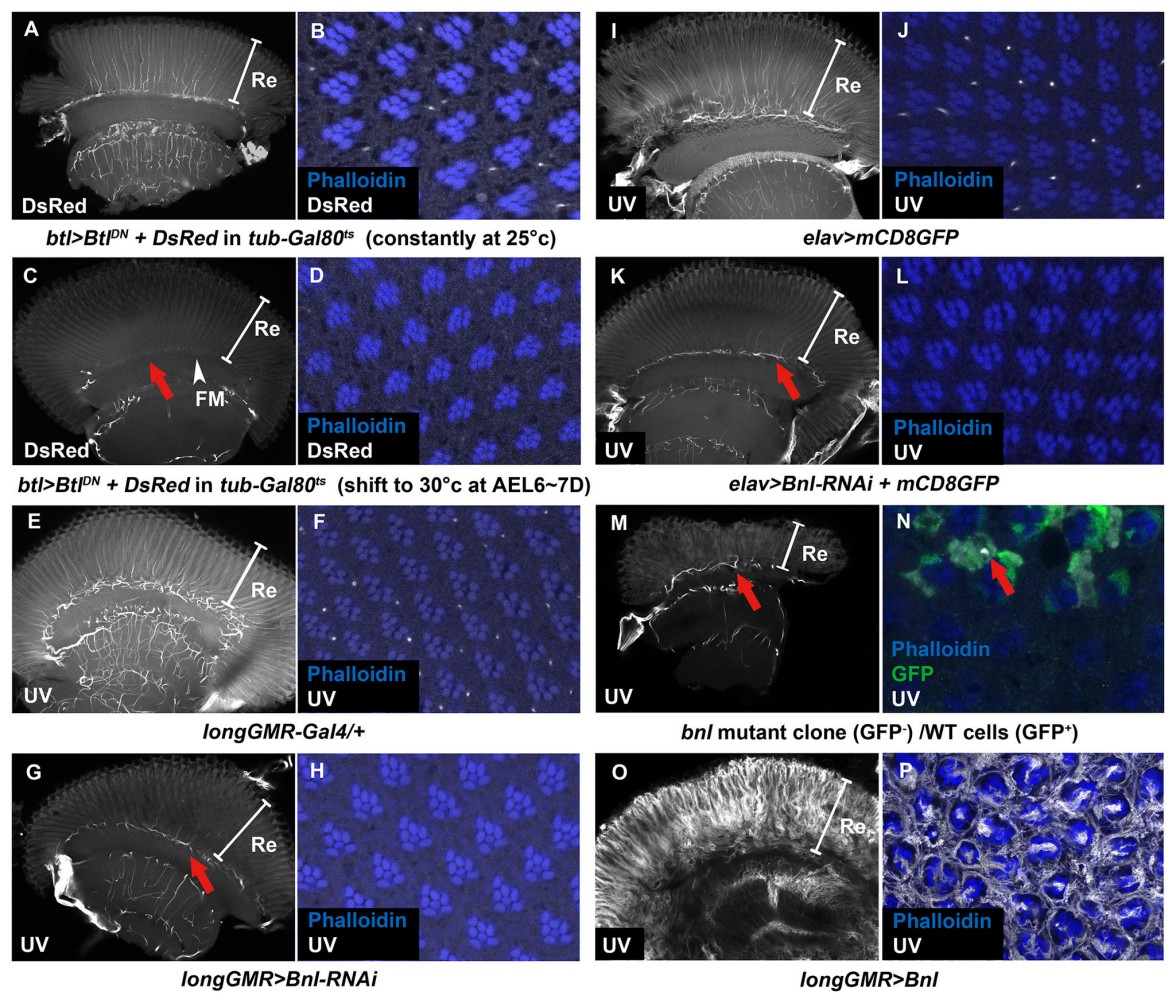
FGF/FGFR signaling control trachea extension in the visual system. The tracheae were labeled by *btl > DsRed* (white in A-D) or UV-excited tracheal autofluorescence (white in E-P). The photoreceptor rhabdomeres were labeled by phalloidin staining (blue) in optical section of whole-mount adult retina. (A–D) Btl is required in the tracheal cells for retinal trachea ingrowth to the retina and the formation of TNUFM. Trachea-specific expression of Btl^DN^ achieved by *btl*>*Btl* ^*DN*^ *+DsRed* in *tub-Gal80* ^*ts*^. (A–B) Flies incubated constantly at 25°C were used as a control, showing normal pattern of retinal trachea. (C–D) Flies incubated at 25°C and shifted to 30°C at 6~7 days AEL, presumably before the air sacs extended branches at pupal stage. Nearly complete loss of trachea and TNUFM (red arrow). (E–H) Bnl is required in retina for the tracheal ingrowth. (E–F) *longGMR-Gal4/+* showed normal patterns of retinal trachea. (G–H) Knockdown of Bnl in retina was achieved by *longGMR>bnl-RNAi* and caused nearly complete loss of retinal trachea. However, the TNUFM seemed normal (red arrow, compare to red arrow in C). (I–J) *elav>mCD8GFP* showed normal patterns of retinal trachea. (K–L) Neuronal specific knockdown of Bnl by *elav>bnl-RNAi+mCD8GFP* caused nearly complete loss of retinal trachea. The TNUFM appeared normal (red arrow). (M–N) Flies with amorphic *bnl* ^00857^/*bnl* ^00857^ *Minute* ^*+*^ mutant clone were generated by *ey-flp*. Wild-type cell were labeled by GFP. (M) A fly eye entirely composed of *bnl* mutant cells (no *w* ^+^ cells) was selected for examination and showed nearly complete loss of retinal trachea. However, the TNUFM seemed normal (red arrow). (N) A fly eye composed partly of *bnl* mutant clones (w^-^) was selected for examination. Trachea was not observed within the clones (non-GFP) but can be found in the wild type region (GFP; red arrow). (O–P) Retina-specific expression of Bnl by *longGMR>Bnl* caused excessive tracheal overgrowth in the retina. Re: retina; FM: fenestrated membrane (arrowhead).

Adult heads with proboscis removed were fixed in 4% paraformaldehyde overnight at 4°C, and washed with 0.3% PBST for 10 min three times. The red eye samples were treated by pigment bleaching process as described above (Figure 4). Before adding the primary antibody, the samples were washed by rocking gently with 0.3% PBST at 4°C overnight, and the washing process was repeated at least two more times to replace the air in the tracheal tubes with 0.3% PBST solution and also washing out the remaining red pigments. This prevents the interference of confocal imaging by light reflection from air-filled tubes and autofluorescence from the retinal pigments. The following primary antibodies were used: rabbit anti-β-Gal (Cappel), mouse anti-Dlg (Developmental Studies Hybridoma Bank, DSHB) and rat anti-Elav (DSHB). Fluorescent secondary antibody conjugates including Cy3, C5 and DyLight series were from Jackson Lab. Phalloidin conjugated with Alexa Fluor series were from Invitrogen. Pupal and adult samples were mounted with FocusClear (CelExplorer Labs Co.) to improve the transparency. For adult whole-mount eye samples, the FocusClear treatment should be more than overnight to ensure penetration. Confocal images were collected by Zeiss confocal system 710 or 510 Meta. 3D reconstructions were processed by ZEN software (Zeiss).

### Transmission electron microscopy

Adult heads with proboscis removed were fixed in fixative (4% paraformaldehyde, 2.5% glutaraldehyde in 0.1M sodium cacodylate, pH7.4) for 2 h, then in 1% tannic acid (Electron Microscopy Sciences) in fixative for another 2 h. After washing six times with 0.1M sodium cacodylate (Sigma), the heads were incubated in 2% OsO_4_ (Electron Microscopy Sciences) in 0.1M sodium cacodylate for 2 h, then washed in water for 10 min three times, and incubated in 2% uranyl acid (Polysciences) overnight. The samples were serially dehydrated with ethanol with 10 min at each step: 50% once, 75% once, 85% once, 95% twice, and 100% three times, then replacement in solution of ethanol/propylene oxide (Electron Microscopy Sciences) with the indicated ratios (3:1, 1:1, 1:3, 0: 1) for 10 min at each step, then in solution of propylene oxide/Spurr (Low Viscosity Embedding Media Spurr’s Kit, Electron Microscopy Sciences) at the same ratios (3:1, 1:1, 1:3, 0: 1) for 2 h at each step. Tissues were then embedded in Spurr’s resin at 70°C for 8 h. Ultrathin sections were sectioned with a diamond knife (Ultracut, Reichert-Jung, Vienna, Austria) and examined by transmission electron microscopy (Tecnai G2 Spirit TWIN, FEI Company, Hillsboro, OR) with a Gatan CCD Camera (794.10.BP2MultiScanTM).

### RNA *in situ* hybridization

*In situ* hybridization of whole-mount dissected pupal eye (58~59% pd) was done with digoxigenin-labeled RNA probe generated from *bnl* Z3-2 full length cDNA [28] as described [42]. Alkaline phosphatase immunohistochemistry was used to visualize *in situ* hybridization signals.

### *tub-Gal80^ts^* temperature-shift condition

The parental flies were crossed at 25°C for three days. The embryos were collected every three hours, incubated at 25°C and shifted to 30°C at 6~7 days (144~168 h) after egg laying (AEL) until eclosion.

## Results

### Tracheal system in the adult *Drosophila* visual system

We used four different methods to observe the trachea in the adult visual system (Figure 1). Mature trachea can be observed by transmitted light using conventional microscopy (Figure 1A), and also by reflected light using confocal microscopy (Figure 1B). These two methods are based on the fact that mature air-filled tracheal tubes can reflect light [9,13,43]. Retinal trachea can also be observed by expressing fluorescent protein using the trachea-specific *btl-Gal4* [24] (Figure 1C, D-D’) and UV-excited tracheal autofluorescence (Figure 1D, D’’) which is dependent on accumulation of the Drop-dead protein in the trachea [44]. These two patterns are almost identical (Figure 1D). In addition, *trachealess* reporter (*trh-lacZ*) expression can be detected in the retinal trachea (Figure 1C, white arrow). The *trh-lacZ* signal showed nuclear localization in the adult (Figure 1C, white arrow) and also in the pupal stage (Figure 3C–H), which is similar to the larval stage [13], in contrast to the cytoplasmic localization in the embryo [45,46]. There are many tracheae present in the region of retina and optic lobe (Figure 1D), but almost no trachea in the region of lamina except the lamina cortex close to the FM at the base of retina (Figure 1D).

The *Drosophila* adult retina is composed of a regular array of about 700-800 ommatidia. One might expect that the retinal trachea would also be distributed in a regular pattern associated with the ommatidia. However, we found that the tracheal distribution in the retina is irregular in both proximal (Figure 2B) and distal regions (Figure 2C) of the retina (rhabdomeres labeled by autofluorescence from red pigment of eye, see also Figure S1). The ratio of ommatidium-to-trachea is about 2:1. Not every ommatidium contact with trachea (Figure 2B-C, Video S1). Some of the retinal tracheae have bifurcating branches (Figure 1D, white arrow). This is in contrast to the observation of one tracheal tube associated with each ommatidium in the house-fly *Musca* *domestica* [47] and blow-fly *Calliphora vicina* [48].

The retinal trachea in the distal region has a dot shaped cross section (Figure 2C, white arrow), while those in the proximal region have a donut-shaped cross section (Figure 2B, white arrow), suggesting that the trachea extends from the proximal part of the retina (close to optic lobe) and into the distal retina where it terminates. The retinal trachea had a diameter less than 1 µm (Figure 2D–F), suggesting that they are tracheoles [49], the finest terminal branches of trachea. The ring structure ( *Taenidium* ), characteristic of tracheal lumen can be observed in the proximal sections by transmission electron microscopy (Figure 2E-F, red arrow). We also observed some mitochondria close to the retinal trachea (Figure 2E-F, blue arrow). This is consistent with the tracheal function in oxygen transport, as mitochondria require oxygen to generate ATP. The mitochondria in ommatidia have also been reported to be predominantly localized to the periphery sites of the photoreceptors [48,50,51], but not in the surrounding pigment cells.

Additionally, we observed a tracheal network under the fenestrated membrane (TNUFM) (thicker tubes in Figure 2A and Video S1). These tubes are thicker than the trachea in the retinal region. The retinal trachea is connected with the TNUFM (Figure 2A, G, K, Video S1). These observations suggest that the tracheal tubes enter the retina through the FM at the base of the retina.

To better understand the tracheal pattern in the adult visual system, we generated a 3D reconstruction from confocal optical sections (Figure 2G, Video S2) and whole-mount head images (Figure 2H-I, K-L). A large air sac, the post-ocular sac (PocSc, based on the size and position [52]. See also Figure 2J) extended several major branches and connected to the TNUFM (Figure 2G, Video S2). Two other air sacs in the head, the post-genal sac (PgeSc) and the dorsal sac (DSc), also connected to the TNUFM (PgeSc: Figure 2H-I, different focal plane in the same sample; DSc: Figure 2K). The number of major branches extending from these air sacs varied. The PocSc had five major branches (Figure 2H-I) while the PgeSc had three major branches (Figure 2H-I) and the DSc had one (Figure 2K). The tracheae in the optic lobe were mainly connected with the PocSc (Figure 2G, Video S2) and also with cervical trachea (CvTra) near the neck (Figure 2L), and are thicker than the tracheal tubes in the retina (Figure 2G, Video S2). The multiple air sacs supporting the visual system imply a high oxygen requirement of the visual system.

### Trachea ingrowth into the eye during the mid-pupal stage

There were no tracheae in the third instar eye-antennal disc (Figure 3A). To understand the development of the retinal trachea, we examined the tracheal pattern in the eye during pupal stages by following *btl*>*GFP* and *trh-lacZ* as markers for tracheal cells. At up to 30% pd, no tracheal cell could be detected in the developing eye (Figure 3B). A group of tracheal cells began to appear at 42% pd (Figure 3C). At 47% pd, two separate air sacs could be observed in the posterior side of the pupal head (Figure 3D). These air sacs are presumably the PocSc (the larger one) and PgeSc (the smaller one). Tracheal lumen can be observed at this stage (Figure 3E, white arrow). At 52% pd, PocSc extended several branches along the basal part of retina, following a posterior-to-anterior direction (Figure 3F–H). But the PgeSc did not extend branches at this time (Figure 3F), indicating that different air sacs may extend branches at different time points. These branches can further bifurcate to form secondary branches (Figure 3H white arrow), presumably eventually forming the TNUFM that was observed in the adult (Figure 2A, Figure 2K). At 60% pd, the trachea can be found extending into the retina in a proximal to distal direction (Figure 3I). Retinal tracheae continue to extend distally following the thickening retina (Figure 3J). The developmental processes of trachea in visual system were summarized in Figure 3K.

### Retinal trachea development depends on FGF/FGFR (Bnl/Btl) signaling

It has been shown that the FGF/FGFR (Bnl/Btl) signaling is important for tracheal extension in embryo and larval stages [10–12,28,53–57]. We examined whether Bnl/Btl signaling also plays an important role in the development of retinal trachea during metamorphosis using a dominant-negative form of Btl (Btl^DN^) driven by the trachea-specific *btl-Gal4*. Constitutive expression of Btl^DN^ in trachea (*btl>Btl* ^*DN*^) has been reported to cause inhibition of tracheoblast formation in the wing disc of third instar larva [12] and also caused late pupal lethality (our study). Therefore we combined *tub-Gal80* ^*ts*^ to block the Gal4 activity in earlier developmental stages. We found that *btl*>*Btl* ^*DN*^ combined with *tub-Gal80* ^*ts*^ at 30°C showed inhibition of tracheoblast formation in wing disc (Figure S2C, white arrow) and also caused pupal lethality. These phenotypes are consistent with the published report [12] and confirmed that the Btl^DN^ is effective and the Gal80^ts^ was non-functional at 30°C. At 25°C, the *btl*>*GFP* signal was strongly repressed in wing disc (Figure S2B; the GFP signal was enhanced to show the weak signal), demonstrating that the Gal80^ts^ was efficient but allowed a low level of leaky expression. These flies showed slightly smaller size of tracheoblast (Figure S2B, white arrow, compare with Figure S2A, white arrow), normal pattern of retinal trachea (Figure 4A-B) and bypassed pupal lethality. These results suggested that the leaky expression allowed by Gal80^ts^ at 25°C was not sufficient to block tracheal development. We then raised the flies at 25°C and shifted to 30°C at mid-pupal stage, the critical time for retinal trachea development established above. The retinal trachea and TNUFM were almost completely abolished (Figure 4C-D). These results suggest that the retinal trachea and the TNUFM both require the Btl receptors in the tracheal cells to receive the directional cue.

Since the Btl receptor is required in the tracheal cells, we expected that the ligand Bnl may be produced from the target field of trachea ingrowth. We knocked down Bnl by *Bnl-RNAi* driven by the eye-specific *longGMR-Gal4* or pan-neuronal *elav-Gal4*. These almost completely abolished the retinal trachea (Figure 4G-H, K-L). In contrast, the control flies showed normal retinal trachea (Figure 4E-F, I-J). The loss of retinal trachea phenotype can also be found in the large amorphic *bnl* mutant clone in the eye (Figure 4M, a fly eye entirely composed of *bnl* mutant cells; Figure 4N, WT cell labeled by GFP while *bnl* mutant cell had no GFP signal). These results suggest that Bnl is required in retinal cells for trachea ingrowth to the retina. However, the TNUFM were not affected in *Bnl-RNAi* knockdown driven by *longGMR-Gal4* (Figure 4G, red arrow), *elav-Gal4* (Figure 4K, red arrow) and also in large *bnl* mutant clone (Figure 4M, red arrow), suggesting that the Bnl signal that guides the TNUFM growth may not be derived from photoreceptors.

In contrast to the results of loss of Bnl/Btl signal, overexpression of Bnl using the eye-specific *longGMR-Gal4* caused excessive growth of retinal tracheae (Figure 4O-P). This suggests that Bnl expressed in retinal cells is sufficient to attract trachea ingrowth to the retina. Together, these results indicate that the retinal trachea development depends on FGF/FGFR (Bnl/Btl) signaling.

### Source of the Bnl ligand

The *longGMR>Bnl-RNAi* knockdown phenotype suggested that Bnl is produced from *longGMR*-expressing cells. Although *longGMR-Gal4* is reported to be more specific to photoreceptors than the regular *GMR-Gal4* [30], we found that it is also expressed in cone cells and primary pigment cells, in addition to photoreceptors (Figure S3). When *Bnl-RNAi* was driven by the neuron-specific *elav-Gal4* (Figure 4 K-L), the retinal trachea was similarly lost as in *longGMR>Bnl-RNAi* (Figure 4 G-H), thus supporting that neurons are the source of Bnl. Further, *elav-Gal4* is also expressed in the interommatidial bristles, presumably in the sensory neurons within the bristles. The *sc* ^10,^ deficiency inactivates two proneural genes achaete (ac) and scute (sc) [58] and causes loss of interommatidial bristles [40], but their pattern of retinal trachea is normal (not shown). Bnl knockdown by *spa-Gal4*, which is expressed in cone cells in the pupal stage [59,60], caused no changes in retinal trachea (not shown). These results suggest that the cone and interommatidial bristles are not the major Bnl-producing cells, and strongly suggest that the photoreceptors are the major Bnl-producing cells. We also tested other cell type-specific Gal4s, including *CG7077-Gal4* (pigment cell-specific) and *repo-Gal4* (glial cell-specific). However, Bnl knockdown by these drivers caused no changes in retinal trachea (not shown). These results supported that pigment cells, cone cells and glia cells are not the major sources of Bnl. In Bnl knockdown by *sca-Gal4* (R8-specific) and in the *sev* ^14^ mutant which has no R7 [38], the pattern of retinal trachea was not affected (not shown). These results suggest that the Bnl is either not produced by R7 or R8 cells, or may be produced by more than one R cell type. Bnl knockdown by *rh1-Gal4* (R1-R6 photoreceptor-specific, but expressed only in the late pupal stage) caused no defect in retinal trachea, consistent with the critical timing of retinal trachea ingrowth to be in mid-pupa stage.

**Figure 5:**
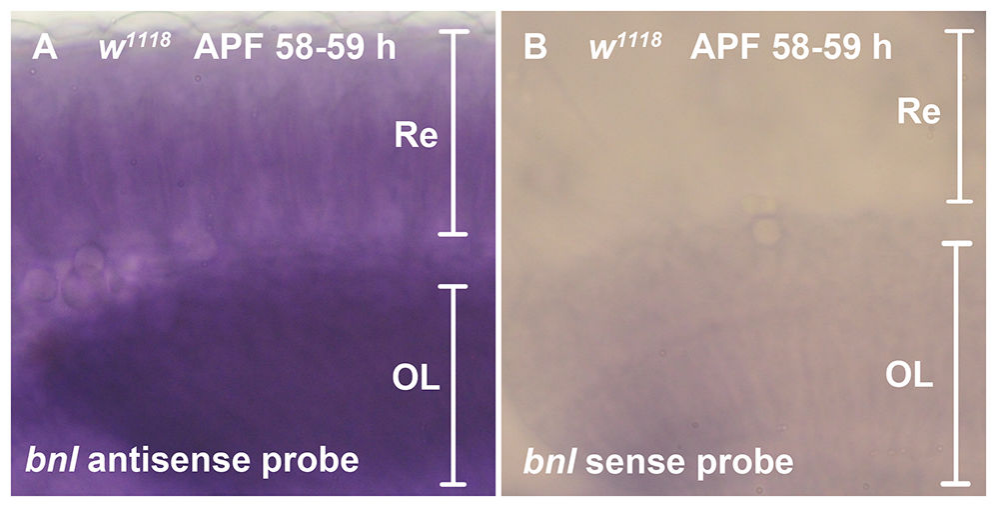
*bnl* expression in pupal eye. RNA *in situ* hybridization detected *bnl* transcripts in the whole-mount dissected eye of 58-59% pd. (A) *bnl* antisense probe was used. Expression in retina (Re) and optic lobe (OL) region can be observed. (B) *bnl* sense probe was used as a negative control. No significant signal can be observed.

The above results led us to predict that Bnl is expressed in photoreceptors. It has been shown that endogenous Bnl protein can be detected as punctates in the cone cells at 28% pd and in the interommatidial bristles at 48% pd [37]. In a *bnl* ^P2^ enhancer trap line [28,61], the *lacZ* reporter expression can be detected strongly in interommatidial bristles (Figure S4A), but not in cone cells (Figure S4A-B) and photoreceptors (Figure S4C), when examined in the eye of 58-59% pd. This is presumably the time when the trachea just before to extend into the retina. A *bnl-Gal4* line [25] showed no significant signal (*bnl>H2B–RFP*) in the eye of 48-55% pd (not shown). Knock-down of Bnl driven by this *bnl-Gal4* (*bnl > Bnl-RNAi*) had no effect on fly development nor retinal trachea development (not shown). Therefore, although the *bnl-Gal4* and *bnl* ^P2^ showed the mimic expression patterns of *bnl* in the embryo [61] and larval wing disc [25], they may not reflect the entire spectrum of *bnl* expression. In order to detect *bnl* expression directly, we performed *bnl* RNA *in situ* hybridization in the pupal eye of 58-59% pd (Figure 5). Expression of *bnl* can be observed in the retina and optic lobe regions, although the spatial resolution did not allow clear distinction of which cell type expressed *bnl*. Combining the RNAi knockdown and *in situ* results, we suggest that photoreceptors are the most likely source of Bnl.

## Discussion

### Multiple stages of tracheal development in the Drosophila visual system

We examined the tracheal pattern in the adult visual system, and then traced its developmental process. Our results showed that there are three major steps for the tracheal development in the visual system. First, air sacs formation occur near the optic lobe in 42-47% pd. Second, in 47-52% pd, these air sacs extend branches along the fenestrated membrane following a posterior-to-anterior direction and further form the TNUFM. Finally, by 60% pd, the TNUFM has extended fine branches into the retina following a proximal-to-distal direction. Thus 52-60% pd is the critical time window for the guidance cues to attract retinal trachea ingrowth.

This late occurrence of the development of the retinal trachea is in contrast to the earlier tracheal development in other imaginal disc-derived structures. For example, the leg trachea appeared at 5 h APF [62], and the notum trachea (dorsal air sac primordium) associated with wing disc appeared at third instar larva [63,64].

### Retinal trachea ingrowth is dependent on FGF/FGFR signaling

Our results showed that the retinal trachea ingrowth requires Btl in the tracheal cells to receive the Bnl signal produced by retinal cells. Overexpression of Bnl in the retina can induce excessive retinal trachea ingrowth. Although we have not directly demonstrated that Bnl is produced by the photoreceptors, a combination of knock-down in different cell types and *bnl in situ* hybridization provides strong evidence that the photoreceptors are the source of Bnl. It has been reported that Bnl/Btl signaling regulated tracheal extension in the embryo [28,53–55,57] and in dorsal air sac primordium associated with larval wing disc [10–12]. Our observation of the developmental process of trachea in the visual system also revealed that the tracheal extension at the second and third stages have different directions (posterior-to-anterior versus proximal-to-distal, respectively). Both stages are affected in tracheal cells expressing *Btl* ^*DN*^, suggesting that the Btl receptor is required in both stages to sense the different directional cues. However, knocking down Bnl in the retinal cells or neurons abolished the retinal trachea which formed at the third stage, but did not affect the TNUFM which formed at the second stage. Thus the same Bnl ligand may be produced by different cells at different stages to provide different directional cues. These results suggest that the Bnl/Btl signaling may be a universal guidance cue for tracheal extension at all developmental stages. However, the source of Bnl can be changed in different developmental stages to regulate the direction of tracheal extension.

### Directionality of guidance cue

The photoreceptor fates become specified in the third instar larval eye disc. They become lengthened during the mid to late pupal stages and the retina becomes thickened [65]. At about the same time, the trachea grows into the developing retina from the base of the retina. One simple hypothesis is that its direction of extension follows the attractive Bnl signal, which may be secreted from the distal side of the thickening retina. It is also interesting that the retinal trachea showed an irregular pattern, rather than a regular relationship to the regular array of ommatidia. Only about half of the ommatidia are associated with a tracheal tube. The position of the tracheal tube is not constant with respect to the ommatidium orientation. This observation suggested that the retinal trachea does not extend along a particular cell type in each ommatidium, therefore the guidance cue may not come from a specific photoreceptor cell type. The relative straight extension of retinal trachea suggested that the extension may be restricted by the physical space permitted by the closely packed ommatidia.

### Developmental regulation versus physiological regulation

The retinal trachea ingrowth occurs at mid to late pupa stage and the extension is unidirectional. The development of such a complex system may be regulated by a hard-wired developmental program, or by physiological conditions that provide temporal and spatial information. The eye develops from the eye disc, which is a flat sheet of two layers of cells, and thus may easily obtain oxygen through its large surface. The retinal trachea ingrowth occurs at mid pupal stage, a time when the photoreceptor cells extend their length and the retina becomes thickened [65,66]. Retina thickness increased from ~35 µm at 55% pd to 100 µm in adult [65]. The photoreceptor rhabdomeres extension start from 37% pd, and reach to the proximal part of the retina at 50% pd [67]. At this time, the tracheal tubes from the TNUFM extend to the fenestrated membrane. Rhabdomeres further extend and the retinal thickness increases in late pupa to adult [65,66]. After 60% pd, tracheae extend into the retina. One interesting possibility is that as the eye grows in size, its ratio of surface area to volume decreases and the photoreceptor cells becomes hypoxic. This hypoxia may induce the expression of Bnl to induce tracheal ingrowth [9]. However, different species of insects show a very broad range of number and morphology of retinal tracheae [18–20,22,23]. The tracheae surrounding the ommatidia (tracheal tapetum) also plays a reflection role for enhancing the light sensitivity of photoreceptors in the nocturnal moth [19]. This is a very different function for the trachea than oxygen supply. These imply another possibility that the retinal trachea may be regulated by developmental cues. The retinal tracheal development in *Drosophila* thus can be an excellent model to test the contribution of physiological conditions on tracheal development versus hard-wired developmental programs in insects.

## Supporting Information

Figure S1 **Autofluorescence from red pigments mark the rhabdomere but not trachea.** (A) The whole-mount dissected *Canton-S* adult eye was excited by 488 nm laser. Autofluorescence of rhabdomere can be detected by GFP emission wavelength. The trachea cannot be detected. (B) The whole-mount dissected eye was excited by 561 nm laser. Autofluorescence of rhabdomere can be detected by weak DsRed emission wavelength. The trachea cannot be detected.
(TIF)

Figure S2 **Temperature-dependent block of Btl signaling by *tub-Gal80*^*ts*^ affected tracheoblast development in larval wing disc.** (A) Tracheal expression of GFP (*btl>GFP*) showed the tracheoblast (white arrow) in a third instar larval wing disc. (B) Tracheal expression of GFP and Btl^DN^ (*btl>GFP+Bnl^DN^*) combined with *tub-Gal80* ^*ts*^, incubated at 25°C constantly (starting from embryo), showed strong repression of the *btl*>*GFP* signal. The GFP signal was enhanced by adjusting the confocal detector in order to observe the tracheoblast (white arrow). The tracheoblast was slightly reduced in size. (C) Tracheal expression of GFP and Btl^DN^ (*btl>GFP+Bnl^DN^*) combined with *tub-Gal80* ^*ts*^, incubated at 30°C constantly (starting from embryo), showed complete repression of tracheoblast formation (white arrow).
(TIF)

Figure S3 **Expression patterns of *longGMR-Gal4* in different developmental stages of retina.** (A-C’’) *longGMR-Gal4* expression patterns were labeled by *longGMR>GFP.nls* (green). (A-A’’) *longGMR-Gal4* expression in cells a few rows behind the morphogenetic furrow in late third instar larval eye disc. Photoreceptors were labeled by anti-Elav (red). (B-C’’) *longGMR>GFP.nls* expression at 50% pd. Nuclei and septate junction were labeled by DAPI (white) and anti-Dlg (red), respectively. (B-B’’) GFP signal can be found in cone cells at the distal level. (B) Four cone cell nuclei can be observed at this optical section (red dotted line). (C-C’’) GFP signal can be found in the photoreceptors at a more proximal level. (C) Photoreceptor nuclei can be observed at this optical section (red dotted line). (D–E) *longGMR-Gal4* expression at the adult stage were labeled by *longGMR>H2B-RFP* (red). Photoreceptor rhabdomeres were labeled by phalloidin staining (blue). (D) RFP signal can be found in the cone cells (c) and primary pigment cells (arrowhead). (E) RFP signal can be found in photoreceptors (eight nuclei surrounding a rhabdomere, white dotted line) as expected.
(TIF)

Figure S4 ***bnl* reporter expression pattern in pupal eye.** (A–C) The *bnl* ^p2^ *lacZ* enhancer trap line was examined in the eye of 58-59% pd to detect the *bnl* expression at the transcriptional level (stained with anti-β-Gal, green). Anti-Dlg (septate junction marker, red) was used to show the cell contours. (A) At the most distal region of ommatidia, *bnl-lacZ* expression can be detected in the interommatidial bristles (red arrowhead). (B) At the cone cells level, there is no expression in the cone cells (c). (C) At a more proximal level, there is no expression in the photoreceptors.
(TIF)

Video S1 **Serial cross-section of retina and its trachea.** Whole-mount dissected adult eye was used for the image. A total of 82 overlapping 1 µm sections (total of 40.5 µm) were compiled into the video. Tracheae were labeled by *btl*>GFP*+DsRed* (yellow). Rhabdomeres were labeled by autofluorescence from the pigments (green). The thicker tubes were the tracheal network under the fenestrated membrane (TNUFM).
(AVI)

Video S2 **3D model of trachea in visual system.** Whole-mount dissected adult eye was used for the image. Tracheae were labeled by *btl > DsRed in GMR-wIR*. 3D model was reconstructed from Z-stacks of longitude sections (230 overlapping 1 µm sections for a total of 146.66 µm thickness). The pseudocolors indicate the depth from periphery (blue) to center (red).
(AVI)
