# Supplementary figures and images for: FGF /FGFR Signal Induces Trachea Extension in the Drosophila Visual System
Source: PLoS One. 2013 Aug 26;8(8):e73878. doi: 10.1371/journal.pone.0073878 (PMC3753266; doi:10.1371/journal.pone.0073878)

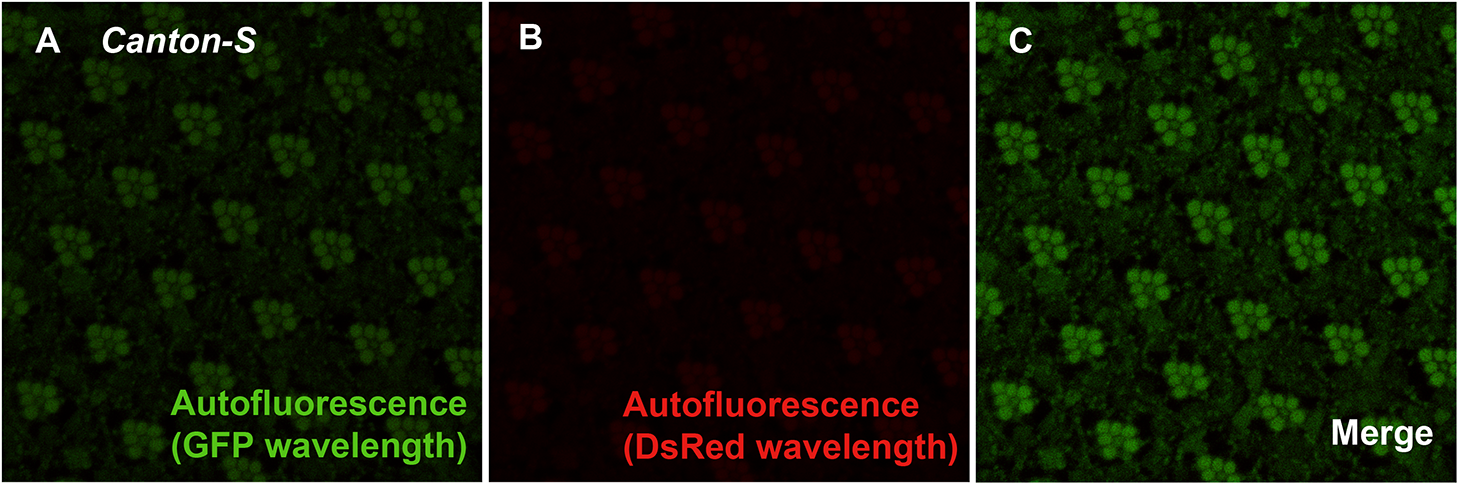

Supplement: Figure S1 — Autofluorescence from red pigments mark the rhabdomere but not trachea. (A) The whole-mount dissected Canton-S adult eye was excited by 488 nm laser. Autofluorescence of rhabdomere can be detected by GFP emission wavelength. The trachea cannot be detected. (B) The whole-mount dissected eye was excited by 561 nm laser. Autofluorescence of rhabdomere can be detected by weak DsRed emission wavelength. The trachea cannot be detected. (TIF) [file pone.0073878.s001.tif]

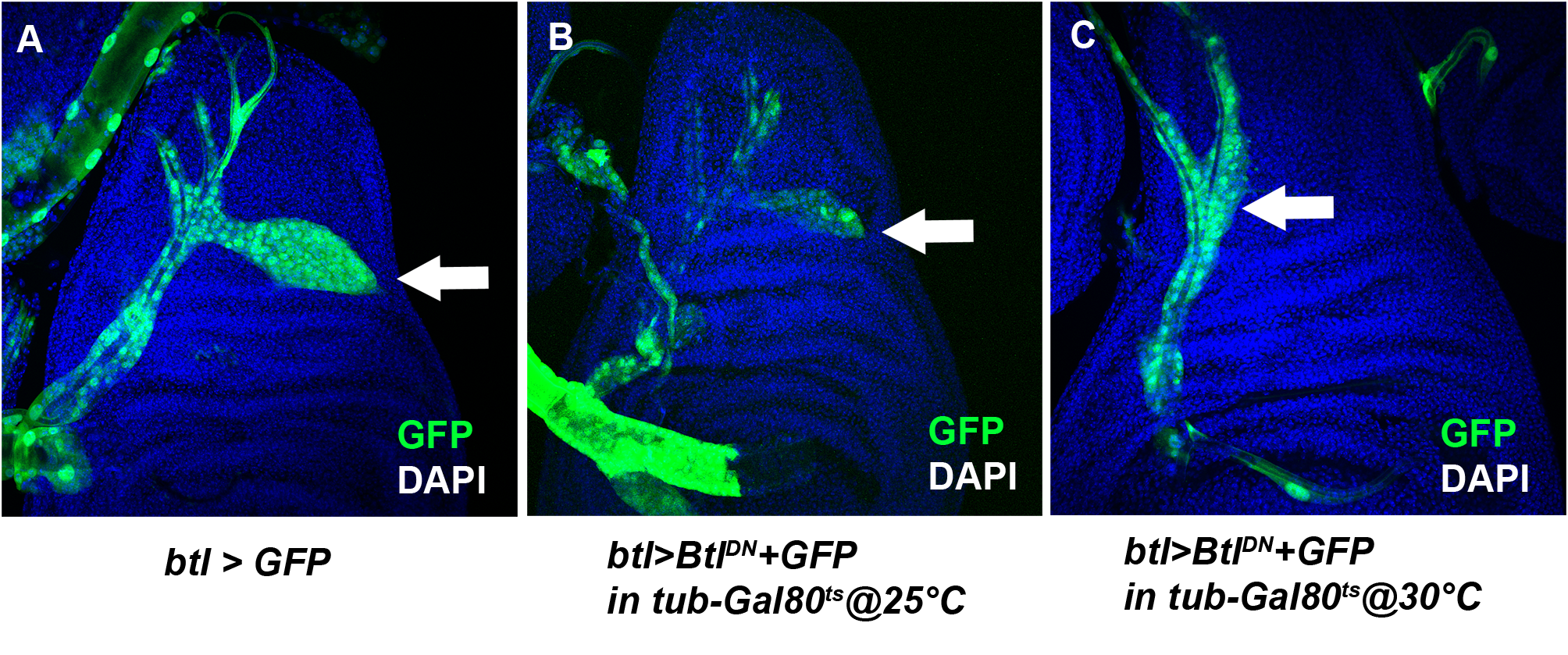

Supplement: Figure S2 — Temperature-dependent block of Btl signaling by tub-Gal80ts affected tracheoblast development in larval wing disc. (A) Tracheal expression of GFP (btl>GFP) showed the tracheoblast (white arrow) in a third instar larval wing disc. (B) Tracheal expression of GFP and BtlDN (btl>GFP+BnlDN) combined with tub-Gal80 ts, incubated at 25°C constantly (starting from embryo), showed strong repression of the btl>GFP signal. The GFP signal was enhanced by adjusting the confocal detector in order to observe the tracheoblast (white arrow). The tracheoblast was slightly reduced in size. (C) Tracheal expression of GFP and BtlDN (btl>GFP+BnlDN) combined with tub-Gal80 ts, incubated at 30°C constantly (starting from embryo), showed complete repression of tracheoblast formation (white arrow). (TIF) [file pone.0073878.s002.tif]

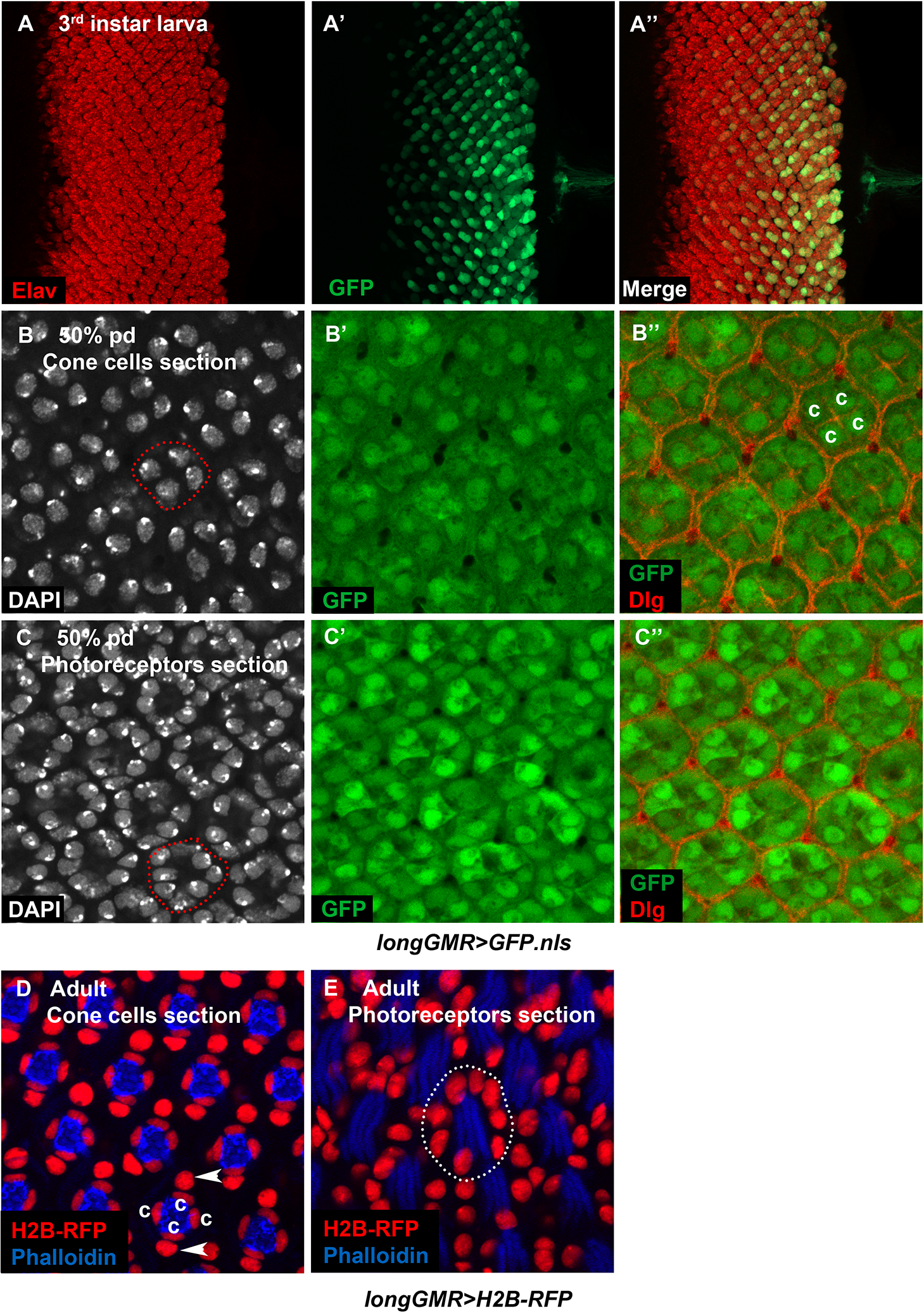

Supplement: Figure S3 — Expression patterns of longGMR-Gal4 in different developmental stages of retina. (A-C’’) longGMR-Gal4 expression patterns were labeled by longGMR>GFP.nls (green). (A-A’’) longGMR-Gal4 expression in cells a few rows behind the morphogenetic furrow in late third instar larval eye disc. Photoreceptors were labeled by anti-Elav (red). (B-C’’) longGMR>GFP.nls expression at 50% pd. Nuclei and septate junction were labeled by DAPI (white) and anti-Dlg (red), respectively. (B-B’’) GFP signal can be found in cone cells at the distal level. (B) Four cone cell nuclei can be observed at this optical section (red dotted line). (C-C’’) GFP signal can be found in the photoreceptors at a more proximal level. (C) Photoreceptor nuclei can be observed at this optical section (red dotted line). (D–E) longGMR-Gal4 expression at the adult stage were labeled by longGMR>H2B-RFP (red). Photoreceptor rhabdomeres were labeled by phalloidin staining (blue). (D) RFP signal can be found in the cone cells (c) and primary pigment cells (arrowhead). (E) RFP signal can be found in photoreceptors (eight nuclei surrounding a rhabdomere, white dotted line) as expected. (TIF) [file pone.0073878.s003.tif]

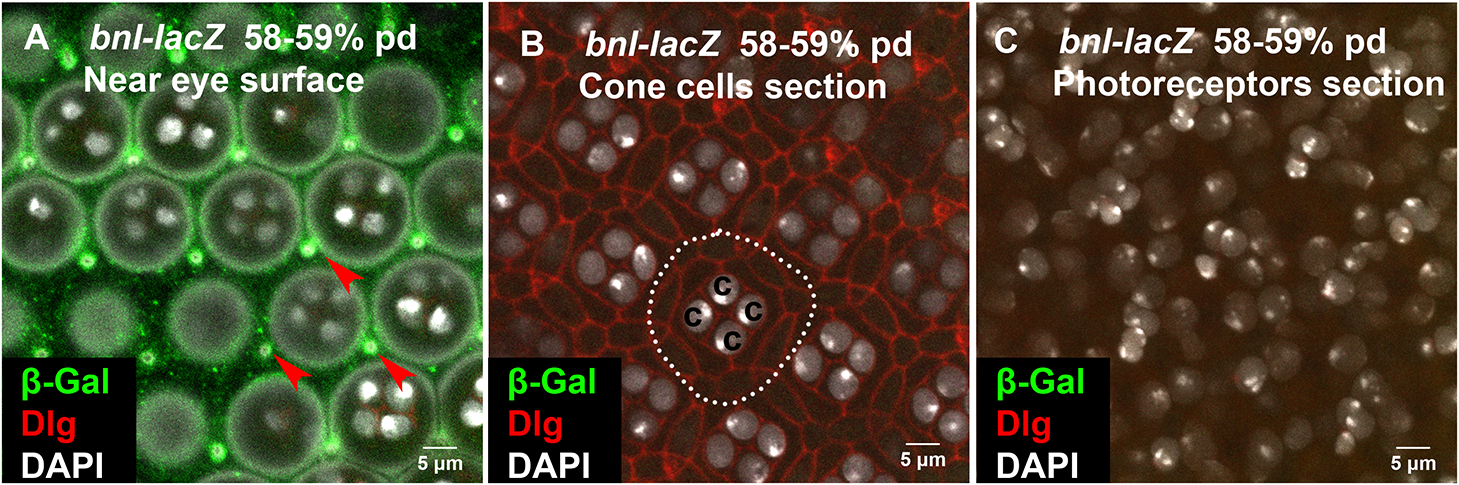

Supplement: Figure S4 — bnl reporter expression pattern in pupal eye. (A–C) The bnl p2 lacZ enhancer trap line was examined in the eye of 58-59% pd to detect the bnl expression at the transcriptional level (stained with anti-β-Gal, green). Anti-Dlg (septate junction marker, red) was used to show the cell contours. (A) At the most distal region of ommatidia, bnl-lacZ expression can be detected in the interommatidial bristles (red arrowhead). (B) At the cone cells level, there is no expression in the cone cells (c). (C) At a more proximal level, there is no expression in the photoreceptors. (TIF) [file pone.0073878.s004.tif]
